# In Vivo Bone Regeneration Induced by a Scaffold of Chitosan/Dicarboxylic Acid Seeded with Human Periodontal Ligament Cells

**DOI:** 10.3390/ijms20194883

**Published:** 2019-10-01

**Authors:** Teerawat Sukpaita, Suwabun Chirachanchai, Pornchanok Suwattanachai, Vincent Everts, Atiphan Pimkhaokham, Ruchanee Salingcarnboriboon Ampornaramveth

**Affiliations:** 1 Research Unit on Oral Microbiology and Immunology, Department of Microbiology, Faculty of Dentistry, Chulalongkorn University, Bangkok 10330, Thailand; kai_m99@hotmail.com; 2 Department of Oral and Maxillofacial Surgery, Faculty of Dentistry, Chulalongkorn University, Bangkok 10330, Thailand; atiphan.p@chula.ac.th; 3 Center of Excellence on Petrochemical and Materials Technology, Chulalongkorn University, Bangkok 10330, Thailand; suwabun.c@chula.ac.th; 4 Bioresources Advanced Materials (B2A), The Petroleum and Petrochemical College, Chulalongkorn University, Bangkok 10330, Thailand; suwattanachai.p@gmail.com; 5 Department of Oral Cell Biology, Academic Centre for Dentistry Amsterdam (ACTA), University of Amsterdam and VU University Amsterdam, Gustav Mahlerlaan 3004, 1081 LA Amsterdam, The Netherlands; v.everts@acta.nl

**Keywords:** chitosan, scaffold, periodontal ligament cells, bone regeneration, calvarial defect

## Abstract

Chitosan/dicarboxylic acid (CS/DA) scaffold has been developed as a bone tissue engineering material. This study evaluated a CS/DA scaffold with and without seeded primary human periodontal ligament cells (hPDLCs) in its capacity to regenerate bone in calvarial defects of mice. The osteogenic differentiation of hPDLCs was analyzed by bone nodule formation and gene expression. In vivo bone regeneration was analyzed in mice calvarial defects. Eighteen mice were divided into 3 groups: one group with empty defects, one group with defects with CS/DA scaffold, and a group with defects with CS/DA scaffold and with hPDLCs. After 6 and 12 weeks, new bone formation was assessed using microcomputed tomography (Micro-CT) and histology. CS/DA scaffold significantly promoted in vitro osteoblast-related gene expression (RUNX2, OSX, COL1, ALP, and OPN) by hPDLCs. Micro-CT revealed that CS/DA scaffolds significantly promoted in vivo bone regeneration both after 6 and 12 weeks (*p* < 0.05). Histological examination confirmed these findings. New bone formation was observed in defects with CS/DA scaffold; being similar with and without hPDLCs. CS/DA scaffolds can be used as a bone regenerative material with good osteoinductive/osteoconductive properties.

## 1. Introduction

After tooth loss, the resorption of the alveolar process is a frequently occurring phenomenon. Many studies have shown that this adversely results in loss of function. Insufficient volume of alveolar bone at the extraction site often impairs the placement of both traditional dentures and dental implants [1,2]. Alveolar ridge preservation methods have been introduced to maintain a sufficient ridge contour in extraction areas. Alveolar ridge preservation can be accomplished by grafting sockets with autografts, allografts, xenografts, and/or alloplasts. However, each of these approaches has its limitations, some are even far from ideal since the material may not successfully be replaced by bone for years. Development of reliable bone tissue engineering materials is therefore crucial for alveolar ridge preservation [3,4,5].

In bone tissue engineering, numerous investigations have shown that 3D porous scaffolds play an indispensable role in controlling osteoblast function and in promoting new bone formation [6,7,8]. The required property of scaffolding material for bone tissue engineering is its osteoconductivity in which osteoprogenitor cells can migrate, adhere, proliferate, and finally differentiate to form bone [9,10]. Seeding of stem cells on properly engineered scaffolds of biocompatible biomaterials was suggested for tissue engineering. Dental mesenchymal stem cells (MSCs), stem cells from human exfoliated deciduous teeth (SHED), dental pulp stem cells (DPSCs), gingival mesenchymal stem cells (GMSCs), and periodontal ligament stem cells (PDLSCs) are popular stem cells used for seeding into 3D scaffolds because they are an easily accessible source of MSCs [11]. Mammana et al. [12] reported the implant of dental human MSCs in a mouse model for treatment of spinal cord injury. They showed that human stem cells can survive and differentiate in different species.

Chitosan has been introduced as a tissue regenerative scaffold because it provides good mechanical support and it promotes cell attachment, proliferation, and differentiation. As a natural-derived product, chitosan has high biocompatibility and therefore meets all criteria to be a good biopolymer for the use in bone and cartilage tissue engineering. Still, the major limitation of chitosan is its limited water solubility. While chitosan scaffolds are considered to be an ideal polymer for making bioactive compounds, there is a potential risk for toxic byproducts from crosslinking agents. The need for utilizing acid and/or chemical reagents as solvents and crosslinkers raises questions about chemical reagent contamination [13,14].

The conventional method to prepare chitosan was to dissolve it in monocarboxylic acids, acids with only one carboxyl group, such as acetic and formic acid. This reaction requires the subsequent utilization of a crosslinking agent such as glutaraldehyde in order to generate the scaffold. This has some disadvantages and therefore the use of other acids has been considered. Besides monocarboxylic acids, there are numerous dicarboxylic acids which are bifunctional organic molecules with two functional carboxyl groups, such as succinic acid and citric acid. Several of these acids are naturally occurring nontoxic solvents and widely used in the food and medicine-related industries. Moreover, multi-carboxylic acids not only solubilize the chitosan but also act as crosslinking agents to improve the mechanical properties of a chitosan scaffold [15,16,17]. Recently, Valderruten et al. [18] reported a novel preparation of chitosan hydrogels using natural occurring dicarboxylic acids as solubilizing and crosslinking agents. This approach greatly reduced the toxicity of chitosan hydrogels. In one of our previous studies [19], we show the use of dicarboxylic acids for preparation of chitosan scaffolds and show the appropriate physicochemical and mechanical properties to use these scaffolds in bone tissue engineering. This material, however, has not been studied for its properties in bone tissue regeneration. In the present study, we evaluated this by using chitosan nano-scaffolds prepared by utilizing dicarboxylic acid as dissolving and cross-linking agent. The scaffold was implanted with or without primary human periodontal ligament cells (hPDLCs) in mouse calvarial defects and formation of bone was analyzed by micro-computed tomography and histology.

## 2. Results

### 2.1. Human Periodontal Ligament Cell Differentiation

**Figure 1:**
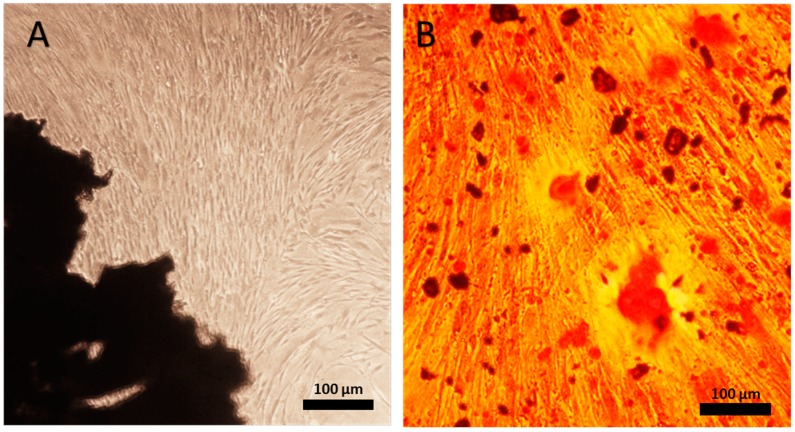
(**A**) Primary periodontal ligament cells (hPDLCs) extracted from periodontal ligament were cultured for 14 days. (**B**) Mineral deposition by hPDLCs after 10 days in osteogenic medium was observed by alizarin red staining. Magnification, ×10 for A; Magnification, ×40 for B. The scale bar represents 100 µm.

Each of 5 isolated hPDLCs cultures showed after plating a typical fibroblastic spindle-shaped morphology (Figure 1A). They reached confluence after 14 days in culture. These 5 populations of hPDLCs were then tested for their osteogenic differentiation potential by a bone nodule formation assay. After being cultured in osteogenic medium for 10 days, the hPDLCs population demonstrating the most intense staining for calcium deposition, resembling mineralization nodules, was chosen for the subsequent in vitro and in vivo study (Figure 1B).

### 2.2. Chitosan/Dicarboxylic Acid (CS/DA) Scaffold Promoted Osteoblast-Related Gene Expression by hPDLCs

**Figure 2:**
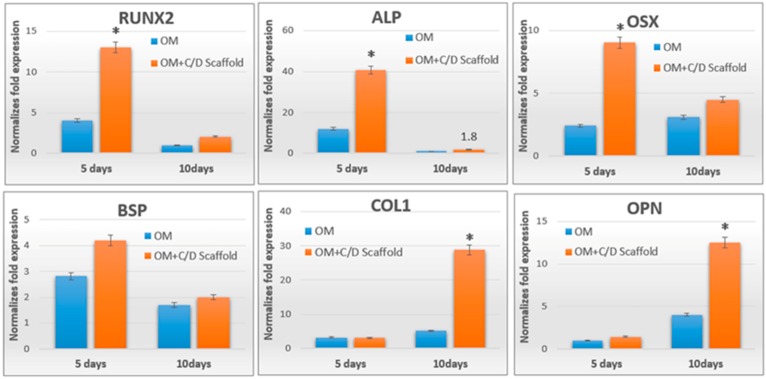
Real-time PCR for osteoblast differentiation associated gene expression, OM: osteogenic medium (independent samples *t*-test between OM group and OM+C/D scaffold group, * *p* < 0.05, *n* = 3).

The chosen population of hPDLCs was cultured with and without CS/DA scaffold in osteogenesis inducing medium for 5 and 10 days, and osteoblast-related gene expression was analyzed. CS/DA scaffold significantly induced the expression of most of the osteoblast-related genes. The time point where the highest expression was found, proved to differ. After 5 days, a marked increase in the level of expression of osteoblast-specific genes, including RUNX2, ALP, and OSX was observed. COL1 and OPN were highest expressed at day 10 in the CS/DA scaffold group (Figure 2). The expression of BSP was not affected by the scaffold.

### 2.3. CS/DA Scaffold Enhanced Bone Regeneration in Calvariae

**Figure 3:**
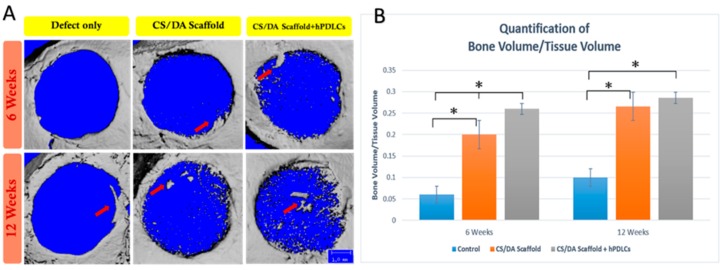
Effect of chitosan/dicarboxylic acid (CS/DA) scaffold on bone regeneration in mouse calvarial defects as assessed by micro-CT scanning. (**A**) New bone formation in mouse calvarial defect (red arrows). (**B**) Quantification of bone volume related to tissue volume (BV/TV). One-way ANOVA, Tukey HSD post hoc test, * *p* = 0.05.

CS/DA scaffolds proved to enhance bone regeneration of calvarial defects of mice. New bone formation was seen both after 6 and 12 weeks in defects implanted with CS/DA scaffold. This was found either with or without hPDLCs. In the control group, the formation of new bone was not found. Newly formed bone was seen throughout the defects as well as at the periphery (Figure 3).

Quantification of bone volume/tissue volume (BV/TV) showed that the amount of newly formed bone at 6 weeks was highest in the CS/DA scaffold with hPDLCs. In this group, significantly more bone was formed than in defects with CS/DA scaffold alone or control defects (*p* < 0.05). After 12 weeks, both CS/DA scaffold groups (with and without hPDLCs) showed a significantly higher amount of newly formed bone than the control group (*p* < 0.05). At this time point, no difference was found between the scaffold alone and the one with cells.

**Figure 4:**
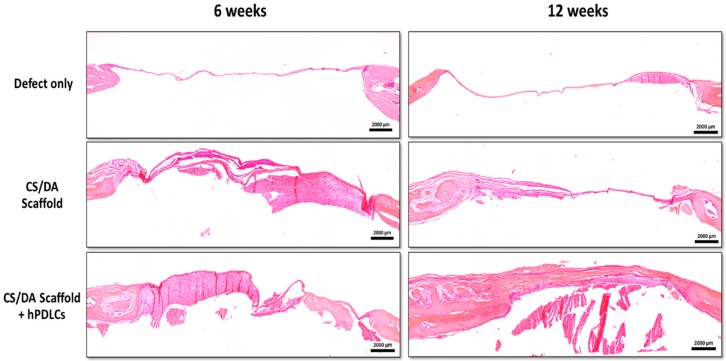
Effect of CS/DA scaffold on bone regeneration in mouse calvarial defects as assessed by H&E staining (magnification ×2)**.**

**Figure 5:**
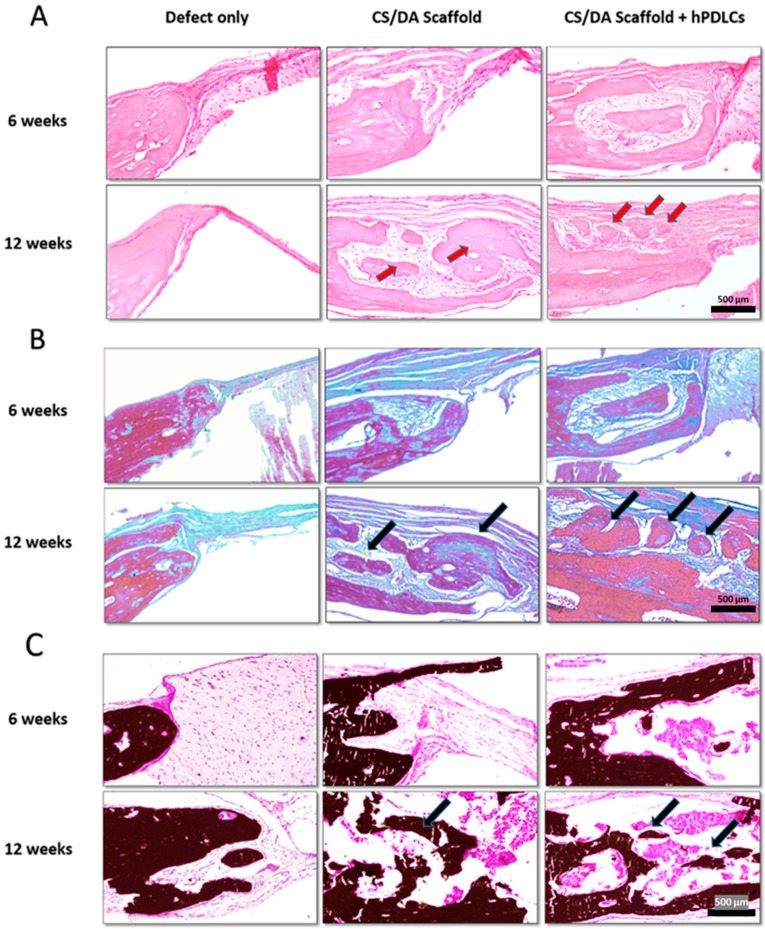
Effect of CS/DA scaffold on bone regeneration in mouse calvarial defects as assessed by histology (×20). (**A**) H&E staining shows newly formed bone and dense connective tissue (red arrows) in the group of CS/DA scaffold implanted with hPDLCs and in the group of CS/DA scaffold alone. (**B**) Masson’s trichrome staining of CS/DA scaffold with and without hPDLCs after 12 weeks showed an increase of blue color representing collagen and mineralized matrix (black arrows). (**C**) Undecalcified sections with Von Kossa staining of both scaffold alone group and scaffold with hPDLCs group showing intense black mass representing mineralized matrix (black arrows).

Histological analysis supported the micro-CT results. H&E stained sections revealed the formation of new bone in defects filled with CS/DA scaffold alone or in those together with hPDLCs (Figure 4; Figure 5A). Masson’s trichrome stained sections revealed an increase in the amount of collagen and bone matrix (represented in blue) in CS/DA scaffold with and without hPDLCs (Figure 5B). Undecalcified sections stained with Von Kossa showed intensely stained mineralized tissue at the margin of the defect (represented in black) resembling bone ingrowth (Figure 5C). It is noticeable that in the control defects without scaffolds no bone formation was found at either time point.

## 3. Discussion

In this study, we demonstrated the regenerative capability of a 3D porous CS/DA scaffold with and without seeded hPDLCs in mouse calvarial defects. We found that the CS/DA scaffold was able to promote bone formation; an effect found either with or without hPDLCs. This is a first report to assess the osteogenic induction potential of a CS/DA scaffold with and without seeded stem cells. Osteogenic differentiation of (stem) cells seeded on a scaffold has been considered a key issue determining the success in new bone formation [20]. Yet, our data demonstrate that also in the absence of cells seeded on the scaffold bone formation occurs.

A drawback of the present study is, however, the use of human cells that were seeded in mice. This method raises the question whether an immune response was evoked. Previous studies, however, have shown that mice do not respond in a negative way to the human cells. In a study comparable to our study, human hPDLCs were shown to be present inside newly formed bone of mouse calvarial defects and the levels of mouse IgG were not affected. The latter report demonstrated that hPDLCs can survive and participate in new bone formation in calvarial defects of the mice [21]. Moreover, other studies showed that stem cells from human exfoliated deciduous teeth had a positive effect on the generation of new bone in mouse calvarial defects. These cells also stained positive for an anti-human-specific mitochondria antibody and were shown to differentiate into osteocytes [22]. Collectively, our findings show, in line with data presented by others, that hPDLCs do not evoke an adverse response in mice and, more importantly, that these cells appear to stimulate initial bone formation in vivo.

Our in vivo data are supported by in vitro findings where we showed a significant increase in CS/DA scaffold-induced expression levels of osteoblast-related genes by hPDLCs. The hPDLCs cultured on CS/DA scaffold showed a marked increase in expression level of RUNX2 and its downstream effector OSX. These genes are considered to be the master genes required for differentiation of osteoblasts and to be early transcription factors for determination of the osteoblast lineage [23]. Other osteoblast-related genes, including COL1, ALP, and OPN, also showed increased levels of expression induced by the CS/DA scaffold. These findings indicate that CS/DA scaffold enhances the differentiation of hPDLCs into osteoblasts.

The osteogenic abilities to stimulate new bone formation of a chitosan scaffold have been reported in a number of studies using different animal models [24,25,26]. Pang and colleagues [27] showed that chitosan solution promoted the synthesis of type I collagen and the differentiation of hPDLCs into osteoblasts. It proved to have the potential to induce new bone formation in calvarial defects of rats. Nandi et al. [28] developed a chitosan scaffold with a controlled release of growth factors including bone morphogenetic protein-2 (BMP-2) and insulin-like growth factor-1 (IGF-1). They showed that this scaffold promoted bone healing and regeneration in a rabbit model. All these data, as well as ours, indicate that chitosan may indeed be an excellent scaffold material for bone formation to occur.

An important issue to consider is where in the defect bone formation occurred. Our data demonstrated that the calvarial defects implanted with CS/DA scaffold both at 6 and 12 weeks show a significant increase in BV/TV. This newly formed bone was generally found both at the margin and in the center of the defect. In particular, the formation in the center of the defect is intriguing. Bone formation at that site indicates that precursors, probably present in the overlying periosteum, were stimulated to differentiate into bone-forming cells. These results indicated that the CS/DA scaffold exhibited osteoconductive and osteoinductive properties. Therefore, the scaffold itself can promote bone regeneration in calvarial defects.

After 6 weeks, scaffolds seeded with cells resulted in a higher amount of bone compared to scaffolds without cells. Such a difference in response was not seen after 12 weeks. These findings indicate a positive effect of the cells during the initial phase of healing. hPDLCs were shown to accelerate the early onset of osteogenesis but appear not to affect bone formation at later time points. 

Our findings indicate that the scaffold material has been lost in a 6-week time interval, at that time point no remnants of the scaffold were found anymore. It is not clear, however, how the in vivo degradation occurs. The scaffold has been removed either by digestion or by dissolving. The latter is unlikely, since in our in vitro experiments, we found that it remained present for at least 10 days when cultured with cells, thus suggesting that the scaffold is not simply dissolved. A possible explanation might be the presence of enzymes which can degrade the scaffold in a time period of six weeks. The observed loss of the scaffold is in line with data presented by Emilia et al. who reported poor long-term stability of pure chitosan being a considerable drawback of the use of chitosan scaffold in tissue engineering applications [29].

Is the loss of the scaffold a negative or positive outcome? In fact, we cannot answer this question. We found that at 12 weeks, which was the latest time point of our in vivo study, bone formation was still very low being only approximately 3% of the defects. Thus, it is clear that complete healing of the skull defects takes far more than 12 weeks [21,30].

## 4. Materials and Methods 

### 4.1. Isolation of hPDLCs

Five molar teeth with healthy periodontal tissues were collected from five healthy 18–25 years-old donors who needed teeth extraction as recommended by their dentist. The procedure was performed under the approval of the Ethical Committee of the Faculty of Dentistry, Chulalongkorn University, Thailand (Approval Number: HREC-DCU 2017-018). Each subject was without systemic and oral infection. The periodontal ligament (PDL) from the middle one-third of molar root surface was scraped off with a surgical blade. The small fragments of PDL were placed in 1 mL culture medium prepared from D-MEM medium added with 1% L-glutamine, 10% FBS, and 1% of antibiotic (#11960, Gibco, Life Technologies Corporation, Grand Island, NY, USA). Subcultures were performed when outgrowing cells reached 80% confluence. These cultures were kept under standard conditions at 37 °C with 5% CO_2_. The 3rd–8th passages of hPDLCs were used in subsequent experiments.

### 4.2. In Vitro Differentiation Assay

To test the osteogenic differentiation potential of the hPDLCs, an in vitro differentiation assay was performed as follows. Five populations of hPDLCs of different donors were cultured to promote osteogenic differentiation. After confluence, the medium was replaced by osteogenic differentiation inducing medium: DMEM containing 10% FBS, 50 μg/mL α-ascorbic acid, 100 mM β-glycerol phosphate, 100 nM dexamethasone, and 1% of antibiotics. The cells were cultured for 10 days. Osteogenic differentiation was examined by staining the formation of mineralized nodules with alizarin red. We selected the hPDLCs line that demonstrated the highest level of mineralization for subsequent experiments.

### 4.3. RT-PCR of Osteogenesis-Related Gene Expression

The expression of osteogenesis-related genes induced by the chitosan scaffold was measured by semiquantitative and real-time RT-PCRs. hPDLCs were cultured in osteogenic medium at a density of 2 × 10^4^ cells/well with and without chitosan scaffold for 5 or 10 day. Total RNA was extracted as previously described [31]. qRT-PCR analysis was performed of the following genes (Table 1): type I collagen (COL1), runt-related transcription factor 2 (RUNX2), osterix (OSX), alkaline phosphatase (ALP), bone sialoprotein (BSP), and osteopontin (OPN).

### 4.4. Preparation of Chitosan/Dicarboxylic Acid (CS/DA) Scaffold

**Figure 6:**
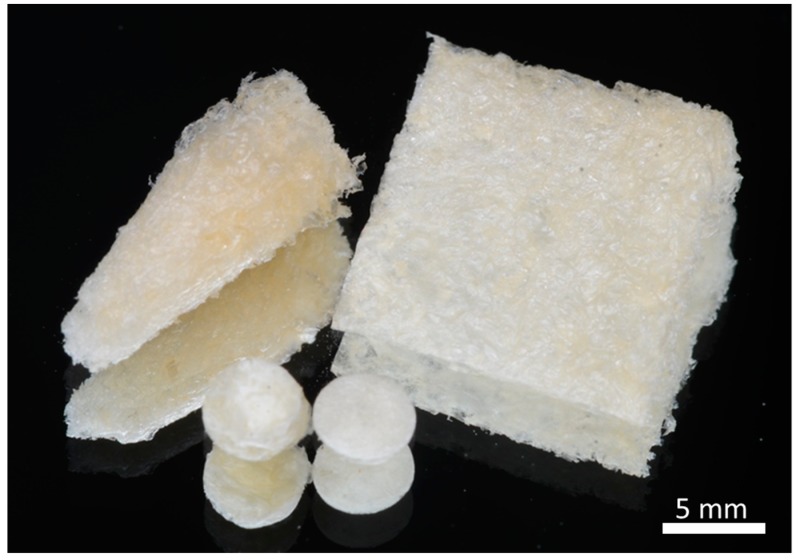
The fabricated CS-DA scaffold after freeze-drying.

Preparation of the scaffold was performed at the Petroleum and Petrochemical College, Chulalongkorn University, Thailand. Briefly, chitosan (85% degree of deacetylation, MW = 500,000) was obtained from Seafresh Chitosan Lab Company Limited (Bangkok, Thailand). Succinic acid (SA, 7.2 mg, 0.06 mmol) was dissolved in deionized water (100 mL) to obtain the SA solution. Chitosan (5 mg, 0.03 mmol) was dissolved in the SA solution (100 mL) to obtain CS-DA 5% w/v. The insoluble fraction of CS was separated by centrifugation. To get the CS-DA hydrogel, 1-(3-dimethylaminopropyl)-3 ethylcarbodiimide hydrochloride (EDC) (65.4 mg, 0.34 mmol) and N-hydroxysuccinimide (NHS) (39.2 mg, 0.34 mmol) were added and dissolved completely before pouring into the tubular mold (1 cm diameter and 1 cm height). The solution obtained was kept overnight at room temperature before washing thoroughly with deionized water and the gel obtained was further dialyzed in deionized water for 1 day. Then, hydrogels were freeze-dried at −20 °C for 36 h to obtain CS-DA scaffold. Finally, the hydrogels were freeze-dried in 5 × 5 mm plastic molds and cut into cylinder-shaped pieces with a biopsy punch (Stiefel, GSK, NC) into samples with a 1 mm height and 4 mm diameter (Figure 6).

### 4.5. Mouse Calvaria Defect Model

**Figure 7:**
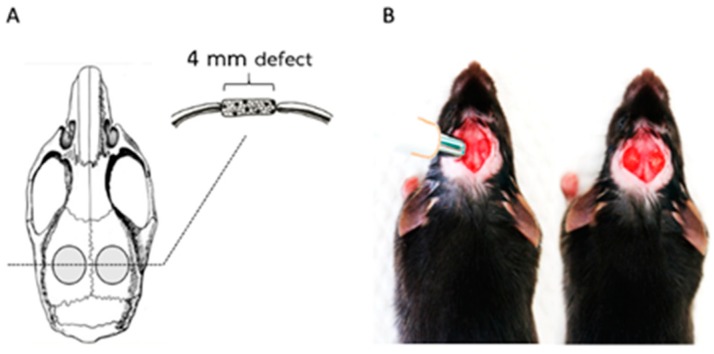
Mouse calvaria defect model. (**A**) Anatomical location of defects. (**B**) Defects were created using a biopsy punch and, in these defects, C/D scaffolds were inserted.

The animal procedure used in this study was modified from the protocol by Spicer et al. [32] and Huynh et al. [22]. The experiment was approved by Chulalongkorn University Animal Care and Use Committee (CU-ACUC), Thailand (Animal Use Protocol No. 1732002). Adult (60-day-old) male C57BL/6Mlac mice (National Laboratory Animal Centre, Mahidol University, Bangkok, Thailand) were used in this study. The sample size was calculated based on the data from our previous similar study [21] using G*Power program, by setting α-error = 0.05, power = 0.8, and effect size = 0.8. For this study eighteen mice were randomly divided equally into 3 treatment groups of 6 each (*n* = 6 mice; 12 defects/group) as follows: (1) defects were filled with Chitosan scaffold, (2) defects were filled with Chitosan scaffold with hPDL cells, and (3) defects were left empty (Figure 7). hPDLCs at a density of 1 × 10^6^ cells/100 μL were seeded on CS/DA scaffolds for 1 h before they were transplanted into the calvarial defects. The mice were anesthetized by an intraperitoneal administration of pentobarbital (NEMBUTAL^®^ Sodium Solution, Akorn, Inc., Lake Forest, IL, USA). The parietal region of the skull was exposed with a 1.5 cm sagittal incision. Two bilateral full-thickness bony defects (4 mm in diameter) were created in the center of each parietal bone by using a biopsy punch (Stiefel, GSK, NC).

### 4.6. Micro-Computed Tomography (Micro-CT)

The mice were euthanized after 6 and 12 weeks. The skulls were fixed immediately in 10% paraformaldehyde for 24 h. Bone formation in the defects was analyzed using micro-CT imaging according to Bouxsein’s guideline [33]. Defects were scanned with an X-ray micro-CT apparatus (SCANCO Medical AG, μCT 35, Switzerland) using established parameter settings for bone tissue. The exposure parameters were 70 kV, 114 μA, and 8 W. The micro CT files were set at a postprocessing threshold using lower and upper threshold values at 241 and 1000. The bone volume and the morphology of the calvarial defects were measured using the manufacturer’s software (SCANCO Medical AG, Switzerland).

### 4.7. Histologic Analysis

After finishing the micro-CT scans, the skull samples were immersed again in paraformaldehyde and dehydrated through a graded series of ethanol. Some specimens were embedded without prior decalcification in methylmethacrylate (ACROS Organics, Geel, Belgium). The samples were embedded in a mixture of Osteo-Bed resin solution containing 2.5 g of benzoyl peroxide per 100 mL at 4 °C for 2 weeks and at 20 °C for 1 day, thus resulting in polymerization. Longitudinal sections of 5 μm were prepared using a microtome (Microm HM355S, Richard Allan Scientific, Kalamazoo, MI, USA). Von Kossa staining was performed to detect mineralized bone structure. The remaining specimens were decalcified with 10% ethylenediaminetetra-aceticacid for 4 weeks and embedded in paraffin. Samples were cut through the center of the defect. Sections of 10 μm thickness were mounted onto individual glass slides and the sections were stained with hematoxylin and eosin (H&E) and Masson’s Trichrome.

### 4.8. Statistical Analysis

The statistical analysis was performed using SPSS statistics software package (SPSS 22.0, IBM, New York, NY, USA). One-way analysis of variance (ANOVA) was used to analyze mineral density of micro-CT data and independent samples comparison *t*-tests were utilized for the data sets of gene expression. Statistical significance was considered at *p* < 0.05. 

## 5. Conclusions

In conclusion, this study has shown that CS/DA scaffolds stimulate bone generation in vivo and that hPDLCs initially promote this process. The scaffolds with and without seeded primary hPDLCs were able to promote bone tissue repair in a critical-size mouse calvarial defect. The CS/DA scaffold could serve as a carrier for stem cells or used alone to repair bone defects and can be used for bone tissue engineering applications.

## Figures and Tables

**Table 1:** The primer sequences of the upstream and downstream primers for these mRNA analyses (5′ to 3′).

Genes	Upstream Primers	Downstream Primers
COL-1	GTGCTAAAGGTGCCAATGGT	ACCAGGTTCACCGCTGTTAC
RUNX2	CCCCACGACAACCGCACCAT	CACTCCGGCCCACAAATC
OSX ALP BSP OPN	GCCAGAAGCTGTGAAACCTC CGAGATACAAGCACTCCCACTTC ATGGCCTGTGCTTTCTCAATG AGGAGGAGGCAGAGCACA	GACAGCAGGGGACAGAAAAG CTGTTCAGCTCGTACTGCATGTC AGGATAAAAGTAGGCATGCTTG CTGGTATGGCACAGGTGATG

## Abbreviations

hPDLCs	Human Periodontal Ligament Cells
CS/DA	Chitosan/Dicarboxylic Acid
